# Mediation Role of Recreational Physical Activity in the Relationship between the Dietary Intake of Live Microbes and the Systemic Immune-Inflammation Index: A Real-World Cross-Sectional Study

**DOI:** 10.3390/nu16060777

**Published:** 2024-03-08

**Authors:** Yanwei You, Yuquan Chen, Mengxian Wei, Meihua Tang, Yuqing Lu, Qi Zhang, Qiang Cao

**Affiliations:** 1 Division of Sports Science & Physical Education, Tsinghua University, Beijing 100084, China; yyw22@mails.tsinghua.edu.cn (Y.Y.); weimx21@mails.tsinghua.edu.cn (M.W.); 2 School of Social Sciences, Tsinghua University, Beijing 100084, China; 13698650651@163.com; 3 School of Public Health and Preventive Medicine, Faculty of Medicine, Nursing & Health Sciences, Monash University, Melbourne, VIC 3004, Australia; cyq199801@126.com; 4 Shanghai Fire Research Institute of Mem, Shanghai 200030, China; yunhua581@163.com; 5 Department of Psychology, Tsinghua University, Beijing 100084, China; 6 Undergraduate Department, Taishan University, Taian 250111, China; 20060136@kust.edu.cn; 7 Department of Earth Sciences, Kunming University of Science and Technology, Kunming 650093, China; 8 School of Pharmacy, Macau University of Science and Technology, Macau 999078, China

**Keywords:** live microbes, recreational physical activity, systemic immune-inflammation index, cross-sectional study, mediation analysis

## Abstract

The main topic of this research is the relationship between dietary intake of live microbe-containing (LMC) foods, recreational physical activity (RPA), and the systemic immune-inflammation index (SII). This study presented a cohort of 26,254 individuals in the National Health and Nutrition Examination Survey (NHANES), representing an estimated weighted population of 193,637,615 in the United States. Weighted multivariable linear regression models were used in consideration of the multi-stage sampling design. Results: The study found that medium-LMC foods were negatively associated with the SII [β (95% CI): −4.807 (−7.752, −1.862), *p* = 0.002], indicating that their intake was correlated with lower levels of the SII. However, no significant associations were found with low- or high-LMC foods. The study also explored the relationship between RPA and the SII, finding that more time spent in RPA was negatively associated with the SII [β (95% CI): −0.022 (−0.034, −0.011), *p* < 0.001]. A mediation analysis was conducted to investigate the role of RPA in the relationship between medium-LMC food intake and the SII. The analysis revealed that RPA had a notable indirect effect, contributing to 6.7% of the overall change in the SII. Overall, this study suggests that medium-LMC food intake and RPA may have beneficial effects on systemic immune inflammation.

## 1. Introduction

Inflammation is a fundamental physiological response to stressors and pathogens [1]. Diet serves as a pivotal modulator of the body’s immune landscape, wielding the ability to either exacerbate or mitigate inflammation [2,3,4]. The incorporation of dietary elements rich in antioxidants (like berries, dark leafy greens, and tomatoes), polyphenols (such as dark chocolate, green tea, and red wine), vitamins (like citrus fruits, carrots, and almonds), and minerals (such as spinach, nuts, and legumes) has been linked to reduced inflammation and enhanced anti-inflammatory processes [5]. The many ways in which nutrients and dietary components shape the immune-inflammatory process are becoming increasingly evident. In recent years, a burgeoning body of research has illuminated the profound impact of dietary components on immune responses and the subsequent modulation of inflammatory markers, as encapsulated by the systemic immune-inflammation index (SII) [6,7,8]. Existing investigations have demonstrated that foods rich in live microorganisms, specifically those containing high levels of live microbes (LMC), such as fermented foods, offer a myriad of health advantages, including anti-inflammatory, antioxidative, anti-microbial, anti-diabetic, and anti-atherosclerotic activities [9]. Hence, the interplay between dietary intake, particularly that of live microbes or probiotics, and the complex realm of inflammation is a subject of burgeoning interest and scientific inquiry. This dynamic relationship underscores the connections between nutrition and immune responses within the human body, and is worthy of further exploration.

Except for diet, physical activity (PA) stands as a potent modulator of immune function, capable of influencing the delicate balance between pro- and anti-inflammatory processes [10,11]. Abundant evidence suggests that regular PA is beneficial for various health outcomes, such as cardiovascular, metabolic, cognitive, and mental health [12,13]. Two distinct categories of physical activity (PA) exist: occupational physical activity (OPA), which encompasses tasks like household chores, and recreational physical activity (RPA). RPA pertains to different forms of exercise during leisure moments, including recreational sports, walking, swimming, cycling, and involvement in fitness classes. Engaging in regular PA has been associated with anti-inflammatory effects, notably through the suppression of pro-inflammatory cytokines and the enhancement of anti-inflammatory mediators [14,15]. One recent study also notes that PA plays an important role in the relationship between the SII and cause-specific mortality [16]. While substantial research has highlighted the favorable effects of overall PA on inflammation, the distinct relationship between RPA and the inflammatory response, particularly concerning the SII, remains unknown.

As noted above, research on the relationship between dietary intake of LMC foods, RPA, and the SII is a captivating realm that merges the realms of exercise physiology and immunology. A deeper comprehension of how physical activity shapes the immune-inflammatory landscape represented by the SII holds the potential to inform strategies for enhancing overall health and attenuating chronic inflammatory conditions. This cross-sectional study aimed to explore the relationship mentioned above using samples from the National Health and Nutrition Examination Survey (NHANES) and investigate whether RPA mediated the associations between the dietary intake of LMC foods and the SII.

## 2. Materials and Methods

### 2.1. Research Design and Participant Inclusion Criteria

The current investigation draws upon the dataset of the National Health and Nutrition Examination Survey (NHANES), a comprehensive and nationally representative cross-sectional survey conducted by the National Center for Health Statistics (NCHS) within the United States. Employing a sophisticated, multistage, probability-based sampling design, the NHANES collects data from the non-institutionalized civilian population and releases this information in two-year cycles. The scope of this analysis encompasses six distinct NHANES data cycles, spanning NHANES 2007–2008 through NHANES 2017–2018, thereby capturing a robust and diverse snapshot of the populace. Prior to participation, all individuals provided written informed consent, and the Research Ethics Review Board at NCHS sanctioned the survey protocol. Constituting a secondary analysis of existing data, this investigation was characterized by the absence of any personal identifiers, obviating the necessity for additional institutional review.

The analysis encompassed a cohort of 34,770 adult participants aged 20 and above. Following this, individuals lacking dietary live microbe-containing (LMC) foods (*n* = 3983) were subsequently excluded, resulting in a refined sample size of 30,787 for subsequent analysis. Further exclusions were then made for participants with incomplete data on blood inflammatory biomarkers (*n* = 1284) and covariates (*n* = 3249). Ultimately, a total of 26,254 participants formed the basis for the ensuing research investigation. All data utilized for this research have been made publicly available through the official NHANES website: http://www.cdc.gov/nchs/nhanes/, accessed on 9 August 2023.

### 2.2. Measurement of Dietary Intake of Live Microbes

This assessment within the framework of the NHANES pertains to the evaluation of the dietary consumption of live microbe-containing (LMC) foods. Utilizing dietary recall interviews, participants were queried about their consumption of foods and beverages known to contain live microbes. The evaluation and quantification of microbial constituents within food products, including those of a fermented nature, were rigorously executed following established methodological frameworks [17,18]. Within the purview of this investigation, the National Center for Health Statistics employed a data integration methodology, harmonizing the 24 h dietary recall data with the US Department of Agriculture Food Surveys Nutrient Database. This synthesis yielded estimations encompassing not only energy intake but also nutrient profiles. A notable endeavor by Sanders et al. warrants special mention, for it underscored the conception and implementation of a systematic framework to enumerate live microbial concentrations per gram across a spectrum of comestibles, numbering a total of 9388 unique codes, further subcategorized into 48 distinct subgroups within the NHANES database.

A systematic classification of food items was undertaken based on their discerned live microbial content. This taxonomy yielded three distinct categories: low microbial content (<10^4^ CFUs/g), medium microbial content (Med; 10^4^–10^7^ CFUs/g), and high microbial content (Hi; >10^7^ CFUs/g). Notably, food items subject to conventional heat treatment procedures, encompassing milk, prepared meat, pork, poultry, seafood dishes, and sauces and gravies, were designated within the “Lo” category, owing to their relatively diminished microbial loads. Conversely, the “Med” category predominantly comprised fresh vegetables and fruits, a reflection of their relatively moderate microbial presence. Fermented dairy products emerged as the principal constituents of the “Hi” category, signifying their heightened microbial abundance.

### 2.3. Measurement of Recreational Physical Activity

Since the NHANES physical activity questionnaire was changed after 2007, the measurement of recreational physical activity (RPA) was self-reported through a detailed questionnaire known as the NHANES Physical Activity Questionnaire. This instrument was specifically designed to assess various aspects of participants’ physical activity behaviors and patterns [19,20]. Participants were asked to provide details such as the type of activity, frequency, duration, and intensity. The questionnaire captured both moderate and vigorous activities and covered a diverse spectrum of exercises, sports, and movement-related behaviors.

The quantification of RPA volume was undertaken employing the unit of minutes per week, a standard metric for such assessments. A calculation methodology was adopted, necessitating participants to delineate the duration spent engaging in RPA during a representative day. Subsequently, the weekly RPA duration materialized through the multiplication of the reported daily duration by the number of days typically allocated to RPA each week. Noteworthy was the utilization of a weighting approach, as proposed by the Physical Activity Guidelines for Americans, which equated 1 min of vigorous recreational activity (VPA) to 2 min of moderate recreational activity (MPA) [21], employing MPA intensity as a standardized metric for gauging the volume of RPA. This recognition of relative intensity informed the procedure, whereby the time allocated to VPA was doubled and integrated with the duration of MPA, thereby culminating in the estimation of total RPA duration.

### 2.4. Measurement of Systemic Immune-Inflammation Index

Quantification of the systemic immune-inflammation index (SII) was undertaken to assess the degree of immune-related inflammation present within the study participants. This index served as an integrative metric, encompassing various blood-based inflammatory markers. These markers included platelet count, lymphocyte count, and neutrophil count, each of which played a role in reflecting the immune response and potential inflammatory processes within the body. Pursuant to the stipulations outlined within the NHANES protocol, the quantification assessment of blood constituents was executed employing the Beckman Coulter methodology. This procedural approach involved the synergistic utilization of an automated dilution and mixing apparatus for sample manipulation, coupled with a singular-beam photometric instrument for hemoglobin-metric evaluations. These techniques were deployed for the enumeration of hematological entities within the blood specimens procured from the Mobile Examination Center (MEC). Drawing from the prior literature, the SII was determined by multiplying the platelet count by the neutrophil count and subsequently dividing by the lymphocyte count [22,23].

### 2.5. Measurement of Covariates

Building upon the groundwork of previous research [20,23], a meticulous selection of covariates was undertaken to ensure a robust analysis. The covariates examined in this study constituted a constellation of factors. Central to this framework were demographic indicators such as age, gender, and race. Moreover, the interplay between socioeconomic status and health outcomes was addressed through the inclusion of marital status, education attainment, and poverty income ratio (PIR). Considering the recognized role of body composition in shaping health trajectories, the covariate of body mass index (BMI) was integrated. The nexus between lifestyle choices and health was illuminated through the incorporation of smoking and alcohol status as covariates, allowing for a more nuanced interpretation of the findings. Acknowledging the impact of chronic diseases, the covariates of diabetes, cardiovascular diseases, and hypertension were included, affording a appraisal of their potential influence on the study outcomes.

### 2.6. Statistical Analyses

The data for analysis were weighted with the requirements of the NCHS. Continuous variables were expressed as means ± standard errors (SE), and categorical variables were represented by percentages (%). We explored the relationship between the dietary intake of LMC foods, RPA, and SII using multivariable linear regression equations. Three discrete models were employed in the analytical framework [23,24]. Model 1 represented the unadjusted configuration, capturing the inherent relationship under examination. Model 2 incorporated adjustments for essential demographic variables, namely age, sex, and race, which were recognized as pivotal determinants in the context of the study. Model 3, the fully adjusted model, extended the adjustments of Model 2 to encompass a broader spectrum of covariates, encompassing body mass index, marital status, education attainment, poverty income ratio, smoking status, alcohol drinking status, diabetes, cardiovascular diseases, and hypertension, thereby accounting for a multifaceted array of potential influences.

Utilizing regression-based mediation analyses, an investigation was undertaken to discern both the immediate impact of adherence to LMC foods on the SII and the subsequent indirect influence mediated through RPA. This examination yielded a triad of estimations: (i) total effect, encapsulating the relationship between adherence to LMC foods and the SII, encompassing both the direct connection and the mediated influence facilitated by RPA engagement; (ii) direct effect, providing a focused elucidation of the association between adherence to LMC foods and the SII; and (iii) indirect effect, unveiling interactions between adherence to LMC foods and the SII, mediated by the involvement in RPA.

The statistical analyses were carried out using R Studio (version 4.2.0) in adherence to robust methodological principles. All statistical tests conducted were characterized by a two-sided nature. A threshold of significance was defined at a *p*-value below 0.05, a convention that underscores the detection of meaningful associations within the confines of the framework under investigation.

## 3. Results

Within the scope of this study, a robust cohort of 26,254 individuals was assembled. This representation was extrapolated to mirror an estimated weighted population of 193,637,615 within the United States. Of these participants, 12,788 (48.71%) identified as male, while 13,466 (51.29%) identified as female, shaping a balanced gender distribution. A detailed exposition of participant attributes, spanning a spectrum of demographic and socio-cultural dimensions, has been documented in Table 1. It is noteworthy that a substantial proportion, reaching 68.79%, self-identified as Non-Hispanic White, signifying a prevailing racial composition within this cohort. Moreover, a significant percentage, constituting 62.16% of the sample, had a college level of education or above. In terms of dietary habits, the average consumption pattern showcased a consumption of 3414 g/d of low-level live microbe-containing (LMC) foods, 106 g/d of medium-level LMC foods, and 23 g/d of high-level LMC foods. Furthermore, the average engagement in recreational physical activity (RPA) revealed an intriguing figure, quantifying at an average of 219.31 min per week, which met the recommendations of the WHO (at least 150 min per week). Systemic immune-inflammation index (SII), a pivotal parameter within our study, registered a mean score of 532 within our population.

Associations between the three types of dietary intake of LMC foods and the SII were detected using weighted linear regression models. In Table 2, it was detected that the intake of medium-LMC foods was negatively associated with the SII in both the unadjusted and adjusted models [Model 1, β (95% CI): −5.690 (−8.777, −2.603), *p* < 0.001; Model 2, β (95% CI): −7.945 (−11.006, −4.885), *p* < 0.001; Model 3, β (95% CI): −4.807 (−7.752, −1.862), *p* = 0.002]. However, as for the low- and high-LMC foods, no significant associations were found in all three models (Tables S1 and S2). This finding suggested that the intake of medium- rather than low- or high-LMC foods was negatively correlated with the SII level.

Furthermore, we explored the relationship between RPA participation and SII (Table 3). As for the crude model (Model 1), more time spent in RPA was negatively associated with the SII [β (95% CI): −0.049 (−0.061, −0.037), *p* < 0.001]. After adjusting for age, sex, and race, the regression coefficient (β) exhibited a value of −0.037 (95% CI: −0.048, −0.025), with a *p*-value below the threshold of statistical significance (<0.001). In the fully adjusted model (Model 3), a similar association was identified [β (95% CI): −0.022 (−0.034, −0.011), *p* < 0.001].

**Figure 1:**
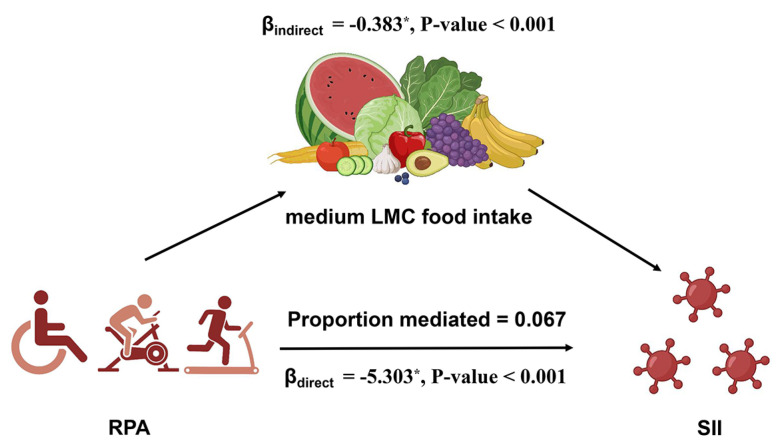
Path diagram of mediation analysis of relationship between RPA, medium-LMC food intake, and SII. * indicates a statistically significance (*p* < 0.05).

A mediation analysis was conducted to explore the role of RPA in the relationship between the dietary intake of medium-LMC foods and the SII (Figure 1 and Table 4). We used Model 3 (fully adjusted model) to conduct the mediation analysis. The total effect on the SII was −5.686 [β (95% CI): −5.686 (−8.222, −3.234), *p* < 0.001], of which the direct effect of medium-LMC foods was −5.303 [β (95% CI): −5.303 (−7.841, −2.807), *p* < 0.001], and the indirect effect of RPA was −0.383 [β (95% CI): −0.383 (−0.586, −0.216), *p* < 0.001]. A notable indirect effect was detected for RPA, contributing to 6.7% of the overall change through the mediating influence of RPA.

## 4. Discussion

This study delves into the relationship between the dietary intake of different LMC foods, RPA, and the SII, scrutinizing 26,254 samples from NHANES 2007–2018. Illuminatingly, we revealed an inverse correlation between medium-LMC foods and the SII. However, no significant observations were identified for low- and high-LMC foods. Subsequently, we detected that RPA level was negatively associated with the SII. Furthermore, our mediation analysis showed that the association between medium-LMC foods and the SII was partly mediated (6.7%) by RPA.

Although the current evidence reports that probiotics found in fermented foods are beneficial to mitigating inflammation [25,26], this study did not detect significant associations between high-LMC foods and the SII. However, the dietary intake of medium-LMC foods, often in the form of fruits and vegetables, was found to be negatively associated with the SII. It is essential to recognize that dietary factors exert a dynamic influence on inflammation through changes in different biomarkers such as interleukin-6 (IL-6), tumor necrosis factor alpha (TNF-α), and C-reactive protein [27,28]. Fruit and vegetables can nurture a balanced gut microbiome, fostering immune homeostasis and quelling inflammation [29,30,31]. The mechanisms encompass gut microbiota modulation, oxidative stress modulation, and the activation of immune cell pathways. These microorganisms, when ingested, interact with the gut microbiota and impact immune regulation. They can stimulate immune cells, such as macrophages and dendritic cells, and influence the production of various cytokines and chemokines, thereby shaping the body’s immune response. The intricate molecular mechanisms underlying the dietary–live microbe–inflammation nexus are multifaceted [32,33]. These mechanisms encompass intricate signaling pathways, such as the Toll-like receptor (TLR) pathways [32,34], which mediate immune responses to microbial stimuli.

Our study identified that more RPA participation was negatively correlated with the SII. Mechanistically, RPA stimulates the release of myokines, which are cytokines secreted by muscles during exercise, exerting systemic anti-inflammatory effects [35,36]. The relationship between RPA and the SII is underpinned by the capacity of exercise to influence immune cell dynamics [15]. Prolonged and vigorous exercise can induce transient inflammation, exemplified by elevated acute-phase reactants. However, with consistent engagement in moderate-intensity exercise, the body gradually adapts, leading to a state of heightened immune surveillance and bolstered anti-inflammatory mechanisms [14]. Consequently, the SII may serve as a dynamic marker reflecting the immune-inflammatory equilibrium shaped by physical activity. Notably, the intricate interplay between RPA and inflammation is bidirectional. Inflammatory processes, particularly those associated with chronic conditions, can impact an individual’s ability to engage in physical activity [37], leading to a potential vicious cycle of reduced exercise tolerance and heightened inflammation.

This research detected a mediation role for RPA participation in the relationship between dietary intake of medium-LMC foods and inflammation. A previous study reported that participants with an anti-inflammatory diet tended to be associated with more RPA [38]. The existing literature has indicated that RPA might serve as a safeguard for the human brain, shielding it from metabolic stress triggered by both postprandial and chronic inflammation [39]. The gut–brain axis and its bidirectional communication between the gastrointestinal tract and the central nervous system would help to explain the modulation of inflammatory responses through this process [40,41]. Regarding the combined impact of RPA and medium-LMC foods on inflammation, a study proposed that supplementation of >1000 mg fruit-derived polyphenols daily for a period of 3 days or more before and after exercise could bolster recovery through mechanisms involving antioxidants and anti-inflammatory properties [42]. Additionally, another investigation underscored the potential of plant-based foods to diminish inflammation markers, suggesting that plant-centered diets could offer safety and performance benefits, particularly in endurance sports [43].

Our study had some strengths and limitations. Primarily, our study harnessed data sourced from a nationwide sample of US adults, thereby enhancing the applicability of our conclusions across broader contexts. Secondly, we considered an array of sociodemographic and health-related variables that might introduce potential confounding influences on the outcomes of our investigation. Despite these strengths, our study was a cross-sectional design, which limited the causal interference as well as analysis of baseline differences. We only evaluated the dietary intake of live microbes without considering the possible impact of gut microbiota. Moreover, it is important to note that while self-reported measures like the NHANES questionnaire provide valuable insights, they may have limitations, including potential recall bias and subjective interpretation. Researchers can combine self-reported data with other objective measures, such as accelerometers, to enhance the accuracy and reliability of the assessment of RPA in the future [44,45]. To comprehensively unravel the relationship between the dietary intake of live microbes, RPA, and the SII, diverse research methodologies are imperative. Further longitudinal studies with repeated measurements assessing how dietary patterns and various forms of exercise influence the SII over time may help to provide crucial insights. Additionally, investigations delving into the mechanistic underpinnings, such as the modulation of immune cell populations and cytokine profiles, may offer a nuanced understanding of the dynamic crosstalk between dietary intake of live microbes, RPA, and inflammation.

## 5. Conclusions

To sum up, this research indicated that (i) groups with higher dietary intake of medium-LMC foods were associated with lower SII; (ii) performing more RPA was associated with lower SII; and (iii) RPA was an important factor to mediate the association between medium-LMC foods and the SII. While research in this field is still evolving, the relationship between dietary intake, RPA, microbial interactions, and inflammation is complex and context-dependent. Factors such as individual variations in microbiota composition, genetics, and the broader dietary context can all influence the outcomes of these interactions. The potential implications for harnessing dietary and exercise strategies to modulate inflammatory responses have far-reaching implications, with promising avenues for preventive and therapeutic interventions in the realm of chronic inflammatory diseases.

## Figures and Tables

**Table 1:** Characteristics of study participants from the National Health and Nutrition Examination Survey (NHANES) 2007–2018.

Category Variables	(%)
Age	
<40	35.78
[40, 60)	37.90
≥60	26.32
Sex	
Male	48.71
Female	51.29
Race/ethnicity	
Non-Hispanic White	68.79
Non-Hispanic Black	10.3
Mexican American	8.09
Other race/ethnicity	12.82
Marital status	
Never married	17.9
Married/living with partner	63.71
Widowed/divorced	18.4
Education	
Below high school	4.79
High school	33.05
College or above	62.16
Poverty income ratio	
<1	14.14
[1, 3)	35.83
≥3	50.03
Body mass index (kg/m^2^)	
<25	29.22
[25, 30)	32.78
≥30	38
Smoking status	
Never smoker	55.65
Former smoker	24.98
Current smoker	19.37
Alcohol status	
Nondrinker	28.48
Moderate alcohol use	51.49
High alcohol use	20.03
Diabetes mellitus	
No	85.57
Yes	14.43
Cardiovascular diseases	
No	91.26
Yes	8.74
Hypertension	
No	62.09
Yes	37.91
**Continuous Variables**	**(Mean ± SE)**
Systemic immune inflammation index (10^9^/L)	532 ± 3.63
Recreational physical activity (minutes/week)	219.31 ± 5.05
Intake of low live microbe-containing foods (100 * g/d)	34.14 ± 0.20
Intake of medium live microbe-containing foods (100 * g/d)	1.06 ± 0.02
Intake of high live microbe-containing foods (100 * g/d)	0.23 ± 0.01

Notes: Weighted percentage (%) for category variables and weighted Mean ± SE for continuous variables. “100 * g/d” represents a unit measured in 100 grams per day of change.

**Table 2:** The association between intake of medium live microbe-containing foods and systemic immune inflammation index.

	β	95% CI	*p*-Value
Model 1			
Intake of medium-LMC foods (100 * g/d)	−5.690	(−8.777, −2.603)	<0.001
Model 2			
Intake of medium-LMC foods (100 * g/d)	−7.945	(−11.006, −4.885)	<0.001
Model 3			
Intake of medium-LMC foods (100 * g/d)	−4.807	(−7.752, −1.862)	0.002

Notes: CI = confidence intervals. Model 1, no covariates were adjusted. Model 2, age, sex, and race were adjusted. Model 3, age, sex, race, body mass index, marital status, education attainment, poverty income ratio, smoking status, alcohol drinking status, and chronic disease conditions were adjusted. LMC foods: live microbe-containing foods. “100 * g/d” represents a unit measured in 100 grams per day of change.

**Table 3:** The association between recreational physical activity and systemic immune inflammation index.

	β	95% CI	*p*-Value
Model 1			
Recreational physical activity (minutes/week)	−0.049	(−0.061, −0.037)	<0.001
Model 2			
Recreational physical activity (minutes/week)	−0.037	(−0.048, −0.025)	<0.001
Model 3			
Recreational physical activity (minutes/week)	−0.022	(−0.034, −0.011)	<0.001

Notes: CI = confidence intervals. Model 1, no covariates were adjusted. Model 2, age, sex, and race were adjusted. Model 3, age, sex, race, body mass index, marital status, education attainment, poverty income ratio, smoking status, alcohol drinking status, and chronic disease conditions were adjusted.

**Table 4:** Mediation pathways among intake of medium live microbe-containing foods, recreational physical activity, and systemic immune inflammation index.

	Estimate	95% CI	*p*-Value
Total effect	−5.686	(−8.222, −3.234)	<0.001
Mediation effect (recreational physical activity)	−0.383	(−0.586, −0.216)	<0.001
Direct effect (intake of medium-LMC foods)	−5.303	(−7.841, −2.807)	<0.001
Proportion mediated (recreational physical activity)	0.067	(0.033, 0.133)	<0.001

Notes: CI = confidence intervals. Model 3 was used. Age, sex, race, body mass index, marital status, education attainment, poverty income ratio, smoking status, alcohol drinking status, and chronic disease conditions were adjusted. LMC foods: live microbe-containing foods.

## Data Availability

Publicly available datasets were analyzed in this study. The dataset presented in this study can be found at https://www.cdc.gov/nchs/nhanes/, accessed on 9 August 2023.

## Supplementary Materials

The following supporting information can be downloaded at: https://www.mdpi.com/article/10.3390/nu16060777/s1, Table S1: The association between intake of low live microbe containing foods and systemic immune inflammation index. Table S2: The association between intake of high live microbe containing foods and systemic immune inflammation index.
