# Supplementary material for: Mediation Role of Recreational Physical Activity in the Relationship between the Dietary Intake of Live Microbes and the Systemic Immune-Inflammation Index: A Real-World Cross-Sectional Study
Source: Nutrients. 2024 Mar 8;16(6):777. doi: 10.3390/nu16060777 (PMC10974920; doi:10.3390/nu16060777)
Supplement: Supplementary file 1 [file nutrients-16-00777-s001.zip › nutrients-2871043-supplementary.pdf]

Table S1. The association between intake of low live microbe containing foods and systemic immune inflammation index.

	β	95% CI	*P*-value
Model 1			
Intake of low LMC foods (100 * g/d)	−0.121	(−0.430, 0.187)	0.437
Model 2			
Intake of low LMC foods (100 * g/d)	0.114	(−0.201, 0.429)	0.473
Model 3			
Intake of low LMC foods (100 * g/d)	−0.104	(−0.423, 0.214)	0.516

Notes: CI = confidence intervals. Model 1, no covariates were adjusted. Model 2, age, sex, race/ethnicity were adjusted. Model 3, age, sex, race, body mass index, marital status, education attainment, poverty income ratio, smoking status, alcohol drinking status and chronic disease conditions were adjusted. LMC foods: live microbe containing foods. "100 * g/d" represents a unit measured in 100 grams per day of change.

Table S2. The association between intake of high live microbe containing foods and systemic immune inflammation index.

	β	95% CI	*P*-value
Model 1			
Intake of high LMC foods (100 * g/d)	−0.442	(−10.136, 9.251)	0.928
Model 2			
Intake of high LMC foods (100 * g/d)	−5.796	(−15.863, 4.271)	0.256
Model 3			
Intake of high LMC foods (100 * g/d)	0.313	(−9.318, 9.944)	0.949

Notes: CI = confidence intervals. Model 1, no covariates were adjusted. Model 2, age, sex, race/ethnicity were adjusted. Model 3, age, sex, race, body mass index, marital status, education attainment, poverty income ratio, smoking status, alcohol drinking status and chronic disease conditions were adjusted. LMC foods: live microbe containing foods. "100 * g/d" represents a unit measured in 100 grams per day of change.
